# Complexity of Frontal Cortex fNIRS Can Support Alzheimer Disease Diagnosis in Memory and Visuo-Spatial Tests

**DOI:** 10.3390/e21010026

**Published:** 2019-01-01

**Authors:** David Perpetuini, Antonio M. Chiarelli, Daniela Cardone, Chiara Filippini, Roberta Bucco, Michele Zito, Arcangelo Merla

**Affiliations:** 1 Institute for Advanced Biomedical Technologies, Department of Neuroscience and Imaging, University G. D’Annunzio of Chieti-Pescara, Via Luigi Polacchi 13, 66100 Chieti, Italy; 2 Department of Medicine and Science of Ageing, University G. d’Annunzio of Chieti-Pescara, Via dei Vestini 31, 66100 Chieti, Italy

**Keywords:** Alzheimer disease, functional near infra-red spectroscopy, signal complexity, clock drawing test, digit span test, corsi block tapping test

## Abstract

Decline in visuo-spatial skills and memory failures are considered symptoms of Alzheimer’s Disease (AD) and they can be assessed at early stages employing clinical tests. However, performance in a single test is generally not indicative of AD. Functional neuroimaging, such as functional Near Infrared Spectroscopy (fNIRS), may be employed during these tests in an ecological setting to support diagnosis. Indeed, neuroimaging should not alter clinical practice allowing free doctor-patient interaction. However, block-designed paradigms, necessary for standard functional neuroimaging analysis, require tests adaptation. Novel signal analysis procedures (e.g., signal complexity evaluation) may be useful to establish brain signals differences without altering experimental conditions. In this study, we estimated fNIRS complexity (through Sample Entropy metric) in frontal cortex of early AD and controls during three tests that assess visuo-spatial and short-term-memory abilities (Clock Drawing Test, Digit Span Test, Corsi Block Tapping Test). A channel-based analysis of fNIRS complexity during the tests revealed AD-induced changes. Importantly, a multivariate analysis of fNIRS complexity provided good specificity and sensitivity to AD. This outcome was compared to cognitive tests performances that were predictive of AD in only one test. Our results demonstrated the capabilities of fNIRS and complexity metric to support early AD diagnosis.

## 1. Introduction

Early Alzheimer’s disease (AD) is characterized by subtle impairments in executive abilities and memory [1,2]. Multiple cognitive test batteries were developed to assess these initial impairments and to allow differential diagnosis between idiopathic dementia and early AD [3,4]. The Clock Drawing Test (CDT) is a functional test that evaluates visuo-constructive and visuo-spatial abilities [5] and it is commonly employed in clinical practice for early dementia screening [6,7]. However, its sensitivity and specificity to early AD is still discussed [8,9]. In fact, novel scoring methods based on CDT outcome were proposed to improve its discrimination capabilities between Mild Cognitive Impairment (MCI) and early AD [5]. The Digit Span Test (DST) and the Corsi Block Tapping Test (CBTT) were developed to assess memory deficits. DST is widely employed in clinics to evaluate short-term memory and verbal working memory abilities [10], whereas the CBTT is employed to estimate visuo-spatial memory skills [11].

Although these tests and associated scorings were developed for sensitive and specific diagnosis of AD, performances in a single test are generally not sufficient for a definitive diagnosis of the disease, often requiring a large set of tests. Functional neuroimaging can be employed during these tests to support diagnosis [12,13]. The importance of using functional neuroimaging during the administration of these tests is widely agreed. In fact, adapted versions of CDT were administrated during a functional Magnetic Resonance Imaging (fMRI) acquisition directly in the MRI scanner [14,15,16]. DST and CBTT were also adapted for functional neuroimaging acquisition (either through fMRI or functional Near InfraRed Spectroscopy, fNIRS) [15,16,17,18,19]. Adaptation could be related to either environmental constraints (because of the fMRI acquisition) or temporal constraints (because of the need of paced tasks that allow standard functional neuroimaging signal analysis). In fact, when performed with concurrent functional brain imaging, these tests are modified from a non-paced administration of tasks and free doctor-patient interaction to a block or event related paradigm where doctor is replaced by an automated stimulation (e.g., through a screen monitor or a minidisk player) [16,17,18,19]. Although these modifications are useful from a neuroimaging standpoint, they indeed alter the ecology of the tests and the interaction between the doctor and the patient. However, these tests characteristics play a primary role in clinical practice and they should be preserved. In this perspective, one of the most suitable neuroimaging techniques is fNIRS that allows functional neuroimaging in outpatient environment. 

fNIRS is a scalp-located non-invasive optical methodology able to record oscillation within the brain of oxygenated (O_2_Hb) and deoxygenated (HHb) hemoglobin related to neuronal activity through the Blood Oxygen Level Dependent (BOLD) effect [20]. This technique is portable, relatively cheap, lightweight and resilient to motion artifacts with a mechanical structure resembling Electroencephalography [EEG] [20] thus being suitable for ecological measurements during the administration of clinical tests. fNIRS was indeed utilized to investigate brain functional alteration in AD during different tasks [21,22,23]. Studies proved the capabilities of fNIRS to investigate functional alterations between AD patients and healthy controls, as well as between AD and MCI patients. fNIRS was also employed during the administration of CDT and DST, however, it was never performed on AD patients and always through a paradigm adaptation. Shoyama and colleagues [24] employed a 52 fNIRS channels system to investigate brain functional activity of healthy subjects during CDT. Statistically significant activations in the superior temporal cortex and in the frontal cortex were detected [24]. Hoshi et al. [25] compared brain functional activation between DST and a modified DST (backward version) and found significant differences in the dorsolateral prefrontal cortex (dorsolateral PFC) [25]. Tian and colleagues [19] employed fNIRS to investigate difference in the brain activation during the administration of DST between patients with post-traumatic stress disorder and healthy controls [19]. Although both groups showed functional activation in the medium PFC, interesting lateralized differences were found. CBTT was administrated together with fNIRS monitoring to 39 healthy participants to clarify the role of the PFC during the task execution [18]. 

Without ad-hoc modifications of the tests structures, the ecological characteristics of these tests definitely do not allow for a standard functional neuroimaging analysis, which requires task-related design matrix to provide statistical inference about brain activity generally based on block averaging or General Linear Model (GLM) [26]. We recently implemented a novel signal analysis approach for functional data acquired through fNIRS that can assess alterations of brain signals in AD overcoming the limitations of standard analysis approaches. In particular, we suggested to estimate the complexity of cortical functional oscillations measured through fNIRS during these tests employing the Sample Entropy (SampEn) metric [27]. SampEn is the negative natural logarithm of the conditional probability that signal subseries of length m (pattern length) that match pointwise within a tolerance r (similarity factor) also match at the m+1 point and it evaluates non-linear predictability of the signal [27]. Moreover, it can be assessed at different time scales using a Multi Scale approach (MSE) [28]. The rationale behind this idea is that AD patients may present a modified structure of functional oscillations during cognitive tests that may reflect in a modified SampEn of the recorded fNIRS signals. SampEn is currently employed for biomedical signal evaluation to assess physiology and pathology. For example, it was used to investigate nonlinear properties of heart rate time series [29,30,31]. Moreover, it was applied to evaluate the complexity of fMRI signals in attention deficit hyperactivity disorder patients [32] and in patients affected by schizophrenia [33]. In AD, it was utilized to analyze resting-state brain activity deriving from Magnetoencephalographic [34] and EEG signals [35,36]. Concerning fNIRS, SampEn was used to investigate cortical activation in AD patients during the administration of Free and Cued Selective Reminding Test [37,38].

In the present study, we report evaluation of fNIRS SampEn in frontal cortex of early AD and healthy controls (HC) during CDT, DST and CBTT as they are performed in clinical practice. Statistical differences in signal complexities between AD and HC were assessed and the capability of fNIRS and SampEn to provide good sensitivity and specificity to the disease was investigated through a multivariate analysis. 

## 2. Materials and Methods 

### 2.1. Participants

Twenty-two participants were recruited in the study. The sample population was composed of eleven early AD patients (mean age ± SD: 72.2 ± 4.5 years; 7 males/4 females) and eleven HC (mean age ± SD: 67.5 ± 5.0 years; 8 males/3 females). The AD patients had a diagnosis of Mild probable Alzheimer’s disease, as defined by the Diagnostic and Statistical Manual of Mental Disorders, 5th edition (DSM-5). Patients with moderate to severe cognitive impairment (Mini Mental State Examination, MMSE < 25/30) [39], vascular dementia, behavioral or psychiatric disorders, hydrocephalus, brain lesions or with a history of stroke or traumatic brain injury were excluded from the study. The study was approved by the Research Ethics Board of the local university, and it was conducted according to the principles described in the Declaration of Helsinki. Informed consent form was signed by all participants before the experiment and they were able to withdraw from it at any time.

### 2.2. Experimental Design

**Figure 1:**
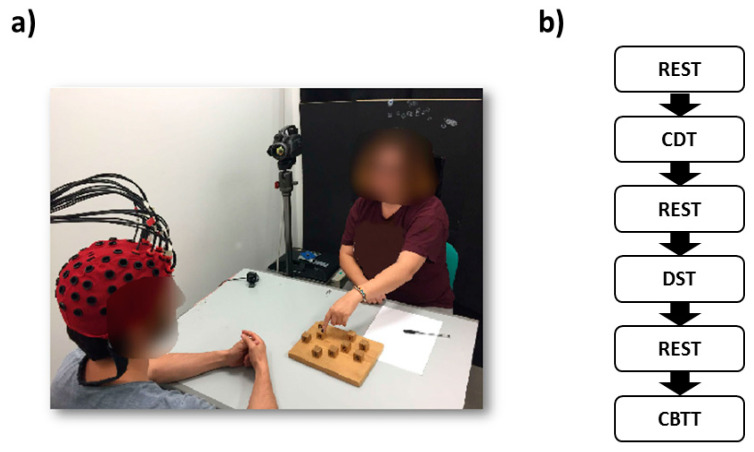
(**a**) Experimental environment and layout. The doctor was sitting in front of the patient while freely interacting with him during the administration of the tests. (**b**) Experimental paradigm. The different tasks, CDT, DST and CBTT were administered to the participants in a consecutively manner, spaced by 1-min rest periods, as they are usually performed in outpatient environment.

Experimental environment and layout are reported in Figure 1. Figure 1a shows the ecological settings of the experiment with the doctor sitting in front of the patient while freely interacting with him during the administration of the tests. Figure 1b shows the experimental paradigm. The different tasks, CDT, DST and CBTT were administered to the participants in a consecutively manner, spaced by 1-min rest periods, as they are usually performed in outpatient environment. Rest periods allowed for avoidance of overlapping effects among tasks. At the start of the experiment, the participants were instructed to relax for 1 min with their eyes closed. Afterward, CDT test started. CDT consisted in presenting to the patients a blank A4-sized paper and in asking to draw a circle with numbers representing a clock. Finally, patients were asked to draw the clock hands at a specific time. After another rest period DST was administered. The patients were asked to repeat a series of digits of increasing length (starting from two) verbally presented by the examiner at a pace of 1 s. If the participants were not able to repeat the sequence, another sequence of the same length was disclosed. The test stopped when the participants could not accurately repeat two series of the same length. After another minute of rest, CBTT was performed. In CBTT, the doctor consecutively tapped an increasing number (starting from two) of cubes located on a wooden tablet (Figure 1a) whose sequence had to be remembered by the patient. The cubes were touched with the index finger at a rate of 1 cube per second. The participants had to tap the cube sequence in the same order immediately after the doctor. The experiment temporal structure resembled that of DST. The overall experiment ended after the administration of CBTT.

### 2.3. Functional Near-Infrared Spectroscopy Instrumentation and Measurement

**Figure 2:**
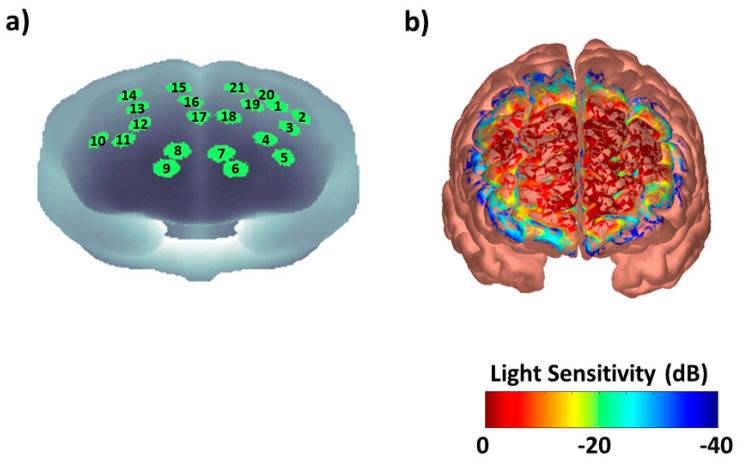
(**a**) Among subjects’ average channels locations after warping of the digitized sources and detectors into MNI space (Colin27). (**b**) Logarithmic channels’ and subjects’ average light sensitivity map displayed up to an attenuation of 100 times (40 dB) showing the frontal and prefrontal sensitivity of the optical probe.

A frequency-domain near-infrared spectroscopy (NIRS) system (Imagent, ISS Inc., Champaign, IL, USA) was used in this study. The system consisted of 32 laser diodes sources, 16 at 690 nm and 16 at 830 nm of wavelength, and 4 photomultiplier-tube (PMT) detectors. The lasers were modulated at 110 MHz and the PMTs at 110.005 MHz for heterodyne detection of modulated light. A time multiplexing scheme was utilized on the sources to prevent cross-talk. The overall instrument sampling rate (accounting for source time-multiplexing) was set at 10 Hz. The light power was lower than 4 mW/cm^2^, in respect of the ANSI standard limits allowing safe measurements. NIR light was carried to the scalp using single optic fibers (0.4 mm core) and from the scalp back to the PMTs using fiber bundles (3 mm diameter). The fibers were located on the frontal and prefrontal areas and were held in place by means of an EEG helmet adapted for NIRS (Figure 1a). The fibers were placed according to the international 10–20 electrode placement system [40]. Source and detector locations were digitized with a Polhemus FastTrak 3D digitizer (Colchester, VT, USA; accuracy: 0.8 mm) using a recording stylus and three head-mounted receivers, which allowed for small movements of the head in between measurements. Optodes layout allowed to measure fNIRS signals from 21 channels (source-detector couple) with source-detector distances separation of either 3 cm or 4 cm. These distances inter-optodes bring to a depth of penetration that allow to measure cortical activity [41]. Figure 2a shows the among subjects’ average channels locations after warping of the digitized sources and detectors into MNI space (Colin27) using AtlasViewerGUI of Homer2 NIRS analysis package [42]. The logarithmic channels’ and subjects’ average light sensitivity map (Jacobian), is displayed in Figure 2b, showing the frontal and prefrontal sensitivity of the optical probe. According to a sensitivity analysis performed in NIRS-SPM [43], the brain Brodmann Areas (BAs) investigated were numbers 8, 9, and 46. Notice that fNIRS is known to investigate regions up to ~3 cm from the scalp surface. Thus, it is indeed suited to investigate superficial cortical regions, as the BAs investigated, not being sensitive to deeper brain structures.

### 2.4. Functional Near-Infrared Spectroscopy Signal Analysis

fNIRS data were analyzed employing a standard continuous wave based fNIRS analysis by means of Homer2 NIRS Processing package [42]. Raw signal intensities were converted into optical densities (ODs). Motion artifacts were identified and removed relying on a wavelet-based algorithm [44]. Artifact free ODs were band-pass filtered using a 3rd order Butterworth filter (0.01–0.4 Hz) to remove slow drifts and physiological contaminations related to heart and breathing rates. ODs were converted into O_2_Hb and HHb oscillations employing the modified Beer-Lambert law (MBLL). In order to further increase the signal to noise ratio (SNR) of functional signals, we employed an algorithm that exploit the brain activity-induced anticorrelation between the two hemoglobin forms (correlation-based signal improvement, CBSI) [45]. CBSI allows to obtain an O_2_Hb signal corrected through HHb. Although this procedure increases O_2_Hb SNR, it creates statistical dependencies among O_2_Hb and HHb, allowing to further analyze only O_2_Hb. Thus, subsequent analysis was performed on O_2_Hb only.

In order to estimate O_2_Hb signal complexity, the SampEn metric was evaluated for each fNIRS channel integrated over the different experimental phases.

The SampEn of a time series {x_1_,…,x_N_} of length N is calculated based on following set of equations [32]:

(1) $$\mathrm{SampEn}\left(m,r,N\right)=-\ln\left[\frac{U^{m+1}\left(r\right)}{U^{m}\left(r\right)}\right]$$

$$U^{m}\left(r\right)={\left[N-\mathrm{mT}\right]}^{-1}\overset{N-\mathrm{mT}}{\underset{i=1}{\sum }}C_{i}^{m}\left(r\right)$$

$$C_{i}^{m}\left(r\right)=\frac{B_{i}}{N-\left(m+1\right)T}$$

$$B_{i}=\mathrm{number}\;\mathrm{of}\;j\;\mathrm{where}\;d\left|X_{i},X_{j}\right|\leq r$$

$$X_{i}=\left(x_{i},x_{i+T}\ldots ,x_{i+\left(m-1\right)T}\right)$$

$$X_{j}=\left(x_{j},x_{j+T}\ldots ,x_{j+\left(m-1\right)T}\right)$$

i≤j≤N−mT, j≠i

where N is total length of the time-series examined, m is the embedded dimension, r is the tolerance factor (scalar for which two subseries with distance below its value are considered identical) and T is the time delay expressed in samples. In this work, the computation of the SampEn was performed choosing a time delay T = 1, an embedded dimension of m = 2 and a similarity factor of r = 0.2·SD, where SD is the Standard Deviation of the signal evaluated. These parameters are commonly employed for complexity analysis of biological signals and they were chosen in accordance with [27].

In order to investigate signal complexity at multiple time scales we further employed the Multiscale Entropy (MSE) method [28]. MSE relies on computing coarse-grained time-series. The course-graining procedure is described by the following equation [32]:

(2) $$y_{j}^{\left(\tau \right)}=\frac{1}{\tau }\overset{j\tau }{\underset{i=\left(j-1\right)\tau +1}{\sum }}x_{i}\;1\leq j\leq \frac{N}{\tau }$$

where $y_{j}^{\left(\tau \right)}$ is the coarse-grained signal for a given sample j and a scaling factor τ which is generated from an average of samples of original signal x. Afterwards, SampEn of each coarse-grained time series is estimated. In the current study only two scale factors of τ = 2, τ = 3 were considered, because of the limited total time of each experimental phase. Moreover, in order to eliminate a possible effect on the SampEn and MSE metric of the time series length, for each experimental phase we cut the last period of each phase for each subject to the shortest one. We obtained homogenized experimental phases lengths for CDT (370 samples), for DST (380 samples) and for CBTT (530 samples).

### 2.5. Statistical Inference and Multivariate Classification

Behavioral results were estimated through unpaired t-tests comparing AD with HC with a NULL hypothesis rejection set at *p* < 0.05. Receiver operating characteristics (ROC) [46] curve was evaluated to provide an estimate of the behavioral tests’ sensitivity and specificity to the disease.

SampEn and MSE for O_2_Hb in each channel, for each experimental phase and each subject were estimated. Average significant differences between AD and HC were tested by means of unpaired t-tests (*p* < 0.05). In order to avoid increased chance of false positive for fNIRS, multiple comparison correction, accounting for channels numerosity, was performed through the False Discovery Rate (FDR) algorithm [47]. Estimation of the BA investigated by the statistically activated channels was performed in NIRS-SPM [43].

A multivariate analysis was implemented to provide a classification of disease (AD or HC), starting from the SampEn and MSE of all the 21 channels in each experimental phase. A linear multiple regression [48] was performed on a dependent variable that labelled the presence of the disease (AD = 1, HC = 0). In order to provide an unbiased estimate of the out-of-sample performance of the classifier a leave one out cross-validation procedure was implemented. This cross-validation procedure consisted in leaving one subject out of the regression and estimating the predicted output value (between 0 and 1) on the given subject. The same procedure was iterated for all the subjects. A ROC curve analysis on the 22 out-of-sample predicted outputs provided estimates of fNIRS signal complexity sensitivity and specificity to the disease in each experimental phase.

## 3. Results

CDT [48] presented significant differences between AD and HC (t = −4.20, df = 20, *p* = 4.4 × 10^−4^). However, DST [49] and CBTT [50] could not discriminate between the two groups (t = −0.31, df = 20, p = N.S., t = −1.45, df = 20, p = N.S.).

**Figure 3:**
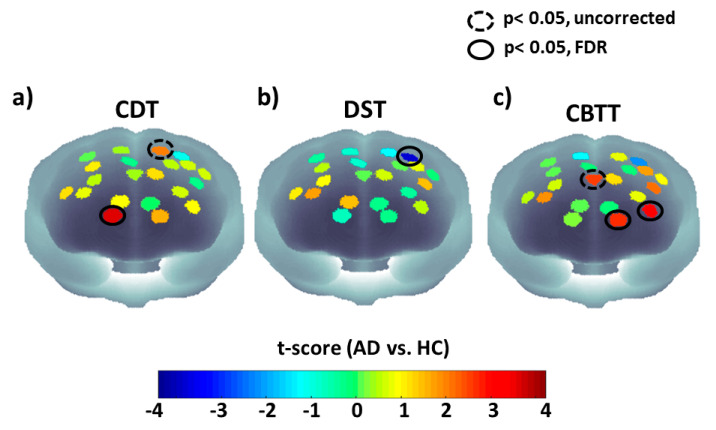
Statistical maps (t-scores) of AD vs. HC for signal complexity of fNIRS for the three experimental phases. Dashed black circles shows significant channels (*p* < 0.05), that did not survive FDR correction, whereas continuous black circles show channels that were still significant (*p* < 0.05) after multiple comparison correction. (**a**) t-score maps (AD vs. HC) based on the SampEn metric during CDT. (**b**) t-score maps (AD vs. HC) based on the SampEn metric during DST. (**c**) t-score maps (AD vs. HC) based on the MSE (τ = 3) metric during CBTT.

Figure 3 reports statistical maps (t-scores) of AD vs. HC for signal complexity of fNIRS for the three experimental phases. Figure 3a,b reports results employing the SampEn metric, whereas Figure 3c reports results employing the MSE (τ = 3).

In the figure we report results where we obtained statistical significance after FDR algorithm. Dashed black circles shows significant channels (*p* < 0.05), that did not survive FDR correction, whereas continuous black circles show channels that were still significant (*p* < 0.05) after multiple comparison correction. A NIRS-SPM-based evaluation of the BA investigated by the significantly activated channels (surviving multiple comparison correction) reported in Figure 3, highlighted higher SampEn for AD in BA 10 for the CDT (Channel 10, t = 3.44, df = 20, *p* = 2.6 × 10^−3^). Significant lower SampEn during the DST was found instead in a channel investigating BA 9 (Channel 20, t = −3.48, df = 20, *p* = 2.4 × 10^−3^). Higher MSE (τ = 3) was finally found in two channels encompassing BA 10 and 46 during CBTT (Channel 4, t = 3.12, df = 20, *p* = 5.4 × 10^−3^, Channel 6, t = 2.63, df = 20, *p* = 0.01). Notice that MSE was estimated for all the different tests. However, CBTT was the only one providing statistically significant results.

**Figure 4:**
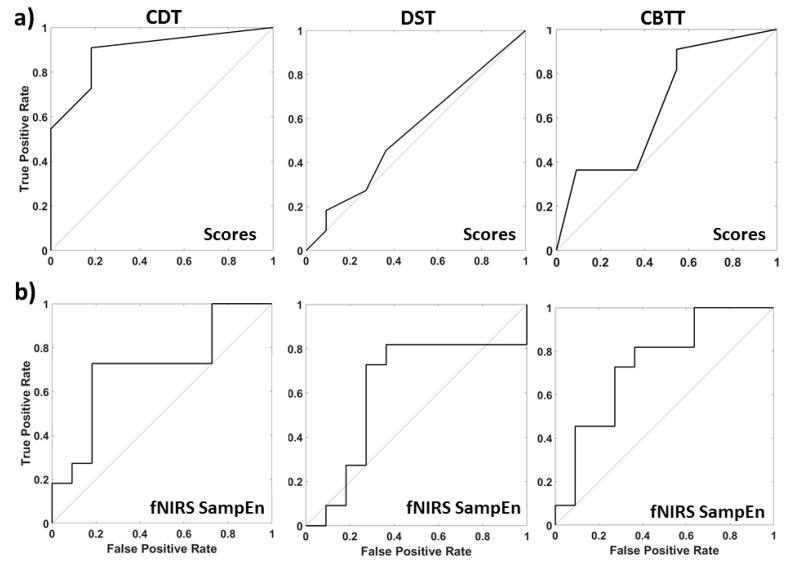
ROC analysis performed on the tests outcome (**a**) and on the fNIRS complexity-based multiple regression analysis (**b**).

ROC analysis performed on the behavioral results and the fNIRS complexity-based multiple regression analysis, described in the method section, are reported in Figure 4. We obtained significant classification outcome when behavioral tests were analyzed only for the CDT (AUC = 0.90) with a best performance sensitivity of 0.82 and specificity of 0.72. DST and CBTT were not able to provide a significant classification (AUC = 0.54 and AUC = 0.60). On the contrary, although less sensitive than CDT test, we obtained above chance classification when relying on fNIRS complexity metric for all the three tests performed with an AUC = 0.75 for CDT, AUC = 0.65 for DST and AUC = 0.71 for CBTT with a best performance sensitivity of 0.73 for all three tests and specificity of 0.82 for CDT and 0.73 for DST and CBTT. Whereas CDT and DST were evaluated on the SampEn metric, CBTT was evaluated on the MSE, which provided channels based significant average differences between AD and HC.

## 4. Discussion

The aim of this study was to assess the capabilities of employing fNIRS during clinical tests that are commonly utilized for AD diagnosis. fNIRS could in fact support these tests by improving sensitivity and specificity to the disease.

Based on our sample population, we found that CDT scores were on average significantly different between AD and HC however DST and CBT were not, further justifying the need of a neuroimaging supporting tool.

In order to preserve the ecological conditions of the tests without altering the standard clinical protocol, we investigated whether a phase-integrated metric of complexity of hemoglobin oscillations could highlight differences between AD and HC. The complexity of the signals was evaluated by means of the SampEn and MSE algorithms.

We found fNIRS channels-based significant AD vs HC differences in SampEn and MSE of O_2_Hb oscillations during CDT, DST and CBTT in brain regions (BAs) involved in visuo-spatial and mnemonic tasks [49]. As demonstrated by Vaillancourt and Newell, these results seem to support the hypothesis that disease can cause a dysregulation of brain physiology that can result in altered functional patterns identifiable through a complexity analysis of time-dependent physiological signals [50]. However, our results do not provide a definitive answer on the direction of the complexity variations due to the disease. Although an increase complexity in some channels was the dominant effect among the different tasks, we indeed had a significant complexity decrease during DST in one fNIRS channel. Previous studies evaluating complexity on electrophysiological brain recordings (EEG [51] and Magnetoencephalography, MEG [34]) showed a decrease in signal complexity with AD, however these studies were performed at rest. On the contrary, signal complexity of fNIRS was here evaluated during the execution of a visuo-spatial and working-memory tests hence strictly evaluating task-related changes induced by AD. Vaillancourt and Newell [50] showed that a pathological state can alter the complexity of an output of a biological system as a function of the input. This input dependent modulation of the alteration makes particularly unpredictable the direction of changes in fNIRS complexity as a function of channel location. In fact, the direction of change might intrinsically depend on the original task-induced activation pattern. This originally unknown activation pattern may indeed justify the variable direction of the complexity changes induced by AD during these clinical tests. In fact, because of the heterogeneous response of different brain region to a given stimulus, fNIRS signals may vary both in space and time. Due to the spatial distribution of fNIRS optodes on the frontal cortex, it is indeed plausible that different channels show a different statistic, generating a peculiar statistical map.

Inferring about the underlying causes of the found fNIRS signal complexity variations induced by AD, early findings by Hock et al. [52] identified reduced oxygenation and cerebral blood flow in AD during the execution of verbal fluency tasks. The authors justified the found alterations in oxygenation and CBF based on an impaired neurovascular coupling. In fact, neurovascular coupling is regulated by means of neurons, glia and vascular cells [51,53,54]. AD patients exhibit a deposition of amyloid β-peptide in neuropil and vessels that could impair such mechanism [55]. Moreover, an altered brain electrical activity and a loss of functional neural connectivity is observed in AD patients [55,56,57], that could further impact a complexity analysis of functional brain modulations.

A ROC curve analysis of clinical tests scores showed above chance classification capabilities only for CDT (AUC = 0.90) which evaluates visuo-spatial skills and apraxia [58]. On the contrary, fNIRS complexity-based multiple regression analysis showed good classification capabilities for all the three tests performed (AUC = 0.75. for CDT, AUC = 0.65 for DST and AUC = 0.71 for CBTT). These results clearly suggest that the use of fNIRS during the administration of cognitive tests may help AD diagnosis.

In fact, in clinical practice it is a common procedure to diagnose AD after long battery of tests, thus intrinsically assuming a high variability in a single test performance and across tests. The group of patients analyzed in this study had in fact a determined diagnosis based on this long battery. Although in our sample CDT seemed particularly suited for a single-test diagnosis, fNIRS entropy-based outcome seemed to provide more stable results among tests compared to cognitive performances. This means that fNIRS seems to be a good supporting tool for a standard multi-test diagnosis.

However, further studies should be performed increasing population sample size. In fact, our study was constituted by a limited number of participants and the fNIRS-based classification outcome, since it relies on a multivariate analysis of all the channels employed, might dramatically increase its performance with a large sample numerosity. In fact, although the study can be considered rather small in sample size, the investigation was conducted employing a leave-one-out cross-validation procedure (eliminating one subject at a time and testing the classifier outcome on that subject), thus intrinsically evaluating the out-of-sample performance. Thus, the results obtained are indeed generalizable. Increasing the sample size may allow further increase of the performance by decreasing a possible in-sample overfitting effect of the classifier.

Further advancement in classification procedure should employ non-linear classifiers, that were not utilizable in this work because of the small sample size and a possible over-fitting effect. Furthermore, it could be of great interest to decouple the contributions of altered brain electrical activity and impaired neurovascular coupling by combining the hemodynamic information provided by fNIRS with electrophysiological signals acquired, for example, by means of synchronous EEG measurements [59] through wearable technology [60]. These brain activity measurements could be further compared with recordings of peripheral autonomic activity (e.g., heart-rate and galvanic skin response monitoring systems) that can be themselves altered by the presence of the disease. Indeed, this study cannot provide an alternative diagnostic tool for early AD, but at least it opens the possibility of utilizing fNIRS as a supporting clinical procedure and it suggests to further explore its diagnostic potentialities.

## 5. Conclusions

In this study, we estimated fNIRS complexity (through SampEn and MSE) in the frontal cortex of early AD and controls during three tests that assess visuo-spatial and short-term-memory abilities (CDT, DST, CBTT). A channel-based analysis of fNIRS complexity revealed AD-induced changes in BAs 9,10 and 46 that where inhomogeneous in direction. A multivariate analysis of fNIRS task-related complexities based on multiple linear regression provided decent specificity and sensitivity to AD. This outcome was compared to test performances that were predictive of AD in one of the three tests (CDT). These findings, although preliminary, seem to confirm the hypothesis that AD may produce a dysregulation of brain electrical activity and neurovascular coupling that may present earlier than clear behavioral impairment. Our results demonstrated the capabilities of fNIRS and complexity metric to support early AD diagnosis.

## References

[1] Dubois B., Feldman H.H., Jacova C., DeKosky S.T., Barberger-Gateau P., Cummings J., Delacourte A., Galasko D., Gauthier S., Jicha G. (2007). Research criteria for the diagnosis of Alzheimer’s disease: Revising the NINCDS–ADRDA criteria. Lancet Neurol..

[2] Baudic S., Dalla Barba G., Thibaudet M.C., Smagghe A., Remy P., Traykov L. (2006). Executive function deficits in early Alzheimer’s disease and their relations with episodic memory. Arch. Clin. Neuropsychol..

[3] Nitrini R., Caramelli P., Porto C.S., Charchat-Fichman H., Formigoni A.P., Carthery-Goulart M.T., Otero C., Prandini J.C. (2007). Brief cognitive battery in the diagnosis of mild Alzheimer’s disease in subjects with medium and high levels of education. Dement. Neuropsychol..

[4] Takada L.T., Caramelli P., Fichman H.C., Porto C.S., Bahia V.S., Anghinah R., Carthery-Goulart M.T., Radanovic M., Smid J., Herrera E. (2006). Comparison between two tests of delayed recall for the diagnosis of dementia. Arq. Neuropsiquiatr..

[5] Ricci M., Pigliautile M., D’Ambrosio V., Ercolani S., Bianchini C., Ruggiero C., Vanacore N., Mecocci P. (2016). The clock drawing test as a screening tool in mild cognitive impairment and very mild dementia: A new brief method of scoring and normative data in the elderly. Neurol. Sci..

[6] Shulman K.I., Pushkar Gold D., Cohen C.A., Zucchero C.A. (1993). Clock-drawing and dementia in the community: A longitudinal study. Int. J. Geriatr. Psychiatry.

[7] Ferrucci L., Cecchi F., Guralnik J.M., Giampaoli S., Noce C.L., Salani B., Bandinelli S., Baroni A., Group F.S. (1996). Does the clock drawing test predict cognitive decline in older persons independent of the Mini-Mental State Examination?. J. Am. Geriatr. Soc..

[8] Pinto E., Peters R. (2009). Literature review of the Clock Drawing Test as a tool for cognitive screening. Dement. Geriatr. Cogn. Disord..

[9] Ehreke L., Luck T., Luppa M., König H.-H., Villringer A., Riedel-Heller S.G. (2011). Clock Drawing Test–screening utility for mild cognitive impairment according to different scoring systems: Results of the Leipzig Longitudinal Study of the Aged (LEILA 75+). Int. Psychogeriatr..

[10] Gerton B.K., Brown T.T., Meyer-Lindenberg A., Kohn P., Holt J.L., Olsen R.K., Berman K.F. (2004). Shared and distinct neurophysiological components of the digits forward and backward tasks as revealed by functional neuroimaging. Neuropsychologia.

[11] Binetti G., Magni E., Padovani A., Cappa S.F., Bianchetti A., Trabucchi M. (1996). Executive dysfunction in early Alzheimer’s disease. J. Neurol. Neurosurg. Psychiatry.

[12] Mueller S.G., Weiner M.W., Thal L.J., Petersen R.C., Jack C.R., Jagust W., Trojanowski J.Q., Toga A.W., Beckett L. (2005). Ways toward an early diagnosis in Alzheimer’s disease: The Alzheimer’s Disease Neuroimaging Initiative (ADNI). Alzheimers Dement..

[13] Aël Chetelat G., Baron J.-C. (2003). Early diagnosis of Alzheimer’s disease: Contribution of structural neuroimaging. Neuroimage.

[14] Formisano E., Linden D.E., Di Salle F., Trojano L., Esposito F., Sack A.T., Grossi D., Zanella F.E., Goebel R. (2002). Tracking the mind’s image in the brain I: Time-resolved fMRI during visuospatial mental imagery. Neuron.

[15] Trojano L., Grossi D., Linden D.E., Formisano E., Hacker H., Zanella F.E., Goebel R., Di Salle F. (2000). Matching two imagined clocks: The functional anatomy of spatial analysis in the absence of visual stimulation. Cereb. Cortex.

[16] Ino T., Asada T., Ito J., Kimura T., Fukuyama H. (2003). Parieto-frontal networks for clock drawing revealed with fMRI. Neurosci. Res..

[17] Kaneko H., Yoshikawa T., Nomura K., Ito H., Yamauchi H., Ogura M., Honjo S. (2011). Hemodynamic changes in the prefrontal cortex during digit span task: A near-infrared spectroscopy study. Neuropsychobiology.

[18] Lancia S., Cofini V., Carrieri M., Ferrari M., Quaresima V. (2018). Are ventrolateral and dorsolateral prefrontal cortices involved in the computerized Corsi block-tapping test execution? An fNIRS study. Neurophotonics.

[19] Tian F., Yennu A., Smith-Osborne A., Gonzalez-Lima F., North C.S., Liu H. (2014). Prefrontal responses to digit span memory phases in patients with post-traumatic stress disorder (PTSD): A functional near infrared spectroscopy study. NeuroImage Clin..

[20] Ferrari M., Quaresima V. (2012). A brief review on the history of human functional near-infrared spectroscopy (fNIRS) development and fields of application. Neuroimage.

[21] Hock C., Villringer K., Müller-Spahn F., Hofmann M., Schuh-Hofer S., Heekeren H., Wenzel R., Dirnagl U., Villringer A. (1996). Near Infrared Spectroscopy in the Diagnosis of Alzheimer’s Disease a. Ann. N. Y. Acad. Sci..

[22] Fallgatter A.J., Roesler M., Sitzmann L., Heidrich A., Mueller T.J., Strik W.K. (1997). Loss of functional hemispheric asymmetry in Alzheimer’s dementia assessed with near-infrared spectroscopy. Cogn. Brain Res..

[23] Herrmann M.J., Langer J.B., Jacob C., Ehlis A.-C., Fallgatter A.J. (2008). Reduced prefrontal oxygenation in Alzheimer disease during verbal fluency tasks. Am. J. Geriatr. Psychiatry.

[24] Shoyama M., Nishioka T., Okumura M., Kose A., Tsuji T., Ukai S., Shinosaki K. (2011). Brain activity during the Clock-Drawing Test: Multichannel near-infrared spectroscopy study. Appl. Neuropsychol..

[25] Hoshi Y., Oda I., Wada Y., Ito Y., Yamashita Y., Oda M., Ohta K., Yamada Y., Tamura M. (2000). Visuospatial imagery is a fruitful strategy for the digit span backward task: A study with near-infrared optical tomography. Cogn. Brain Res..

[26] Friston K.J., Holmes A.P., Worsley K.J., Poline J.-P., Frith C.D., Frackowiak R.S. (1994). Statistical parametric maps in functional imaging: A general linear approach. Hum. Brain Mapp..

[27] Richman J.S., Moorman J.R. (2000). Physiological time-series analysis using approximate entropy and sample entropy. Am. J. Physiol.-Heart Circ. Physiol..

[28] Costa M., Goldberger A.L., Peng C.-K. (2005). Multiscale entropy analysis of biological signals. Phys. Rev. E.

[29] Kim W.-S., Yoon Y.-Z., Bae J.-H., Soh K.-S. (2005). Nonlinear characteristics of heart rate time series: Influence of three recumbent positions in patients with mild or severe coronary artery disease. Physiol. Meas..

[30] Al-Angari H.M., Sahakian A.V. (2007). Use of sample entropy approach to study heart rate variability in obstructive sleep apnea syndrome. IEEE Trans. Biomed. Eng..

[31] Lake D.E., Richman J.S., Griffin M.P., Moorman J.R. (2002). Sample entropy analysis of neonatal heart rate variability. Am. J. Physiol.-Regul. Integr. Comp. Physiol..

[32] Sokunbi M.O., Fung W., Sawlani V., Choppin S., Linden D.E., Thome J. (2013). Resting state fMRI entropy probes complexity of brain activity in adults with ADHD. Psychiatry Res. Neuroimaging.

[33] Sokunbi M.O., Gradin V.B., Waiter G.D., Cameron G.G., Ahearn T.S., Murray A.D., Steele D.J., Staff R.T. (2014). Nonlinear complexity analysis of brain fMRI signals in schizophrenia. PLoS ONE.

[34] Gómez C., Hornero R., Abásolo D., Fernández A., Escudero J. (2009). Analysis of MEG background activity in Alzheimer’s disease using nonlinear methods and ANFIS. Ann. Biomed. Eng..

[35] Abásolo D., Hornero R., Espino P., Alvarez D., Poza J. (2006). Entropy analysis of the EEG background activity in Alzheimer’s disease patients. Physiol. Meas..

[36] Escudero J., Abásolo D., Hornero R., Espino P., López M. (2006). Analysis of electroencephalograms in Alzheimer’s disease patients with multiscale entropy. Physiol. Meas..

[37] Perpetuini D., Bucco R., Zito M., Merla A. (2017). Study of memory deficit in Alzheimer’s disease by means of complexity analysis of fNIRS signal. Neurophotonics.

[38] Lemos R., Simões M.R., Santiago B., Santana I. (2015). The free and cued selective reminding test: Validation for mild cognitive impairment and A lzheimer’s disease. J. Neuropsychol..

[39] Folstein M.F., Folstein S.E., McHugh P.R. (1975). “Mini-mental state”: A practical method for grading the cognitive state of patients for the clinician. J. Psychiatr. Res..

[40] Homan R.W., Herman J., Purdy P. (1987). Cerebral location of international 10–20 system electrode placement. Electroencephalogr. Clin. Neurophysiol..

[41] Chiarelli A.M., Maclin E.L., Low K.A., Mathewson K.E., Fabiani M., Gratton G. (2016). Combining energy and Laplacian regularization to accurately retrieve the depth of brain activity of diffuse optical tomographic data. J. Biomed. Opt..

[42] Huppert T.J., Diamond S.G., Franceschini M.A., Boas D.A. (2009). HomER: A review of time-series analysis methods for near-infrared spectroscopy of the brain. Appl. Opt..

[43] Ye J.C., Tak S., Jang K.E., Jung J., Jang J. (2009). NIRS-SPM: Statistical parametric mapping for near-infrared spectroscopy. Neuroimage.

[44] Molavi B., Dumont G.A. (2012). Wavelet-based motion artifact removal for functional near-infrared spectroscopy. Physiol. Meas..

[45] Cui X., Bray S., Reiss A.L. (2010). Functional near infrared spectroscopy (NIRS) signal improvement based on negative correlation between oxygenated and deoxygenated hemoglobin dynamics. Neuroimage.

[46] Zweig M.H., Campbell G. (1993). Receiver-operating characteristic (ROC) plots: A fundamental evaluation tool in clinical medicine. Clin. Chem..

[47] Genovese C.R., Lazar N.A., Nichols T. (2002). Thresholding of statistical maps in functional neuroimaging using the false discovery rate. Neuroimage.

[48] Neter J., Kutner M.H., Nachtsheim C.J., Wasserman W. (1996). Applied Linear Statistical Models.

[49] Catani M., Dell’Acqua F., Bizzi A., Forkel S.J., Williams S.C., Simmons A., Murphy D.G., de Schotten M.T. (2012). Beyond cortical localization in clinico-anatomical correlation. Cortex.

[50] Vaillancourt D.E., Newell K.M. (2002). Changing complexity in human behavior and physiology through aging and disease. Neurobiol. Aging.

[51] Deng B., Liang L., Li S., Wang R., Yu H., Wang J., Wei X. (2015). Complexity extraction of electroencephalograms in Alzheimer’s disease with weighted-permutation entropy. Chaos.

[52] Hock C., Villringer K., Müller-Spahn F., Wenzel R., Heekeren H., Schuh-Hofer S., Hofmann M., Minoshima S., Schwaiger M., Dirnagl U. (1997). Decrease in parietal cerebral hemoglobin oxygenation during performance of a verbal fluency task in patients with Alzheimer’s disease monitored by means of near-infrared spectroscopy (NIRS)—Correlation with simultaneous rCBF-PET measurements. Brain Res..

[53] Girouard H., Iadecola C. (2006). Neurovascular coupling in the normal brain and in hypertension, stroke, and Alzheimer disease. J. Appl. Physiol..

[54] Croce P., Zappasodi F., Merla A., Chiarelli A.M. (2017). Exploiting neurovascular coupling: A Bayesian sequential Monte Carlo approach applied to simulated EEG fNIRS data. J. Neural Eng..

[55] Pijnenburg Y.A.L., Vd Made Y., Van Walsum A.V.C., Knol D.L., Scheltens P., Stam C.J. (2004). EEG synchronization likelihood in mild cognitive impairment and Alzheimer’s disease during a working memory task. Clin. Neurophysiol..

[56] Stam C.J., Jones B.F., Nolte G., Breakspear M., Scheltens P. (2006). Small-world networks and functional connectivity in Alzheimer’s disease. Cereb. Cortex.

[57] Supekar K., Menon V., Rubin D., Musen M., Greicius M.D. (2008). Network analysis of intrinsic functional brain connectivity in Alzheimer’s disease. PLoS Comput. Biol..

[58] Pasquier F. (1999). Early diagnosis of dementia: Neuropsychology. J. Neurol..

[59] Chiarelli A.M., Zappasodi F., Di Pompeo F., Merla A. (2017). Simultaneous functional near-infrared spectroscopy and electroencephalography for monitoring of human brain activity and oxygenation: A review. Neurophotonics.

[60] Chiarelli A.M., Libertino S., Zappasodi F., Mazzillo M.C., Di Pompeo F., Merla A., Lombardo S.A., Fallica G.P. (2017). Characterization of a fiber-less, multichannel optical probe for continuous wave functional near-infrared spectroscopy based on silicon photomultipliers detectors: In-vivo assessment of primary sensorimotor response. Neurophotonics.

